# Caring About Dogs, Filming Dogs, Watching Dogs: How Far Does the Human–Dog Relationship Travel Through Cultural Production? A Case Study of *Good Boy* (2025)

**DOI:** 10.3390/ani16142168

**Published:** 2026-07-13

**Authors:** Nancy Rebout, Audrey Besegher, Sara Hoummady

**Affiliations:** 1 UniLaSalle, IDEALISS ULR 7519, 76130 Mont-Saint-Aignan, France; sara.hoummady@unilasalle.fr; 2 LECD, Université Paris Nanterre, 200 Avenue de la République, 92000 Nanterre, France; 3 Agir pour la Vie Animale, Association Agir pour la Vie Animale, 40 Le Quesnoy, 76220 Cuy-Saint-Fiacre, France

**Keywords:** animal welfare in media, human–dog relationship, canine behavior, horror movie

## Abstract

Western societies increasingly treat dogs as sentient family members, but does this reshape cultural production, and can cultural production, in turn, reshape the relationship? We traced this circuit through *Good Boy* (2025), a horror film with a real, untrained dog as the sole protagonist, combining filmmaker interviews, behavioral coding of eleven sequences (21-behavior repertoire, permutation tests), and thematic analysis of 242 reviews on the Letterboxd website. The filmmaker restructured the production around his dog’s welfare, yet signs of stress persisted. The film’s canine perspective generated formal innovation but remained confined to human perception. Audiences showed concern for the real dog but read his discomfort as devotion—no review mentioned any behavioral indicator of stress. *Good Boy* reveals not the failure of a single film but the current state of a cultural transition—a society that has moved beyond treating dogs as props yet lacks the tools to act on its own values. Addressing this gap will require welfare monitoring during production, representation that goes beyond human perception, and public literacy in canine body language.

## Simple Summary

Nowadays, people see dogs as family members—but does that change how they are treated in movies? The horror movie *Good Boy* (2025) was examined as a case study by coding the dog’s on-screen body language, analyzing filmmaker interviews and social media reviews. The filmmaker adapted the production to Indy’s needs. Yet Indy still exhibited various signs of unease. The film tried to show the world through a dog’s eyes, but the result stays closer to how humans imagine a dog’s world than to how dogs experience it. Viewers worried about Indy but saw his stress as loyalty—not a single review spotted any sign that he was uncomfortable. Society cares about dogs more than ever but still lacks the right tools: clear welfare rules for filming, better ways to represent their experience, and a basic understanding of what dogs are communicating.

## 1. Introduction

Animals have occupied a central place in cinema since its origins, serving as performers, narrative catalysts, and symbolic mediators of human–animal relations [1,2]. Yet their presence has long been marked by ethical asymmetry. In the early decades of filmmaking, animals were frequently treated as expendable resources subordinated to the demands of realism and spectacle. The injuries and deaths of numerous animals during early cinematic productions highlighted a profound lack of regulation within the industry, initiating a complex historical critique of animal exploitation in media [3]. A striking historical example of these early systemic risks is the production of *Ben-Hur* (1925) [4], where approximately one hundred horses reportedly lost their lives during the filming of the famous chariot race, a tragedy documented by film historian Kevin Brownlow [5]. This event stands as an emblematic milestone in the history of screen cruelty, echoing the broader ethical arguments raised by film scholars such as Barbara Creed (2014) [6]. Contemporary welfare science has since moved beyond the prevention of physical harm: integrative frameworks now recognize that animal well-being encompasses affective experience, behavioral expression, and agency [7,8]. Regulatory systems, however, have not kept pace. Enforcement remains uneven and primarily oriented toward overt physical harm, leaving broader questions—training methods, affective states during production, post-production outcomes—unaddressed [9,10].

But cinema does not only affect the animals appearing in movies. It also shapes how society perceives, desires, and treats them. The use of chimpanzees in entertainment has been shown to distort public perception of their endangered status [11]. In the experimental design, participants exposed to commercials featuring chimpanzees in anthropomorphic settings showed a reduced understanding of the species’ endangered status. Donations to a conservation charity were also lowest in the entertainment conditions, suggesting that the distortion extends beyond perception to behavioral commitment. The popularized “Nemo effect” is often invoked to argue that exposure to fictional characters triggers surges in demand for specific species; in reality, Militz and Foale (2017) [12] found little evidence of such a demand surge in import/export data, arguing that the perceived effect is largely a media narrative that may itself undermine conservation messaging. Clearer evidence of a media-driven shift in demand comes from dogs: Weir and Kessler (2022) [13] found that American Kennel Club registrations rose significantly for breeds portrayed as heroes and fell for those portrayed as anthropomorphized for up to five years after a film’s release. The authors note that such portrayals may carry welfare implications, since they could leave owners with unrealistic expectations of breeds whose behavioral needs are demanding. Film classification systems, however, are built to assess harm to human audiences, while on-set monitoring such as the American Humane Association’s addresses only physical harm to animals during filming; neither currently treats the representational harm a film can do to an entire species [14]. Cinema thus operates at the intersection of on-set ethics and symbolic power: it shapes not only how animals are treated during filming but also how they are perceived, cared for, or abandoned within society.

Horror cinema is a particularly useful genre for examining this dynamic because it is built on the boundary between the familiar and the threatening. Non-human animals have always sat on that boundary. In horror, animals typically serve one of two functions: they are either monstrous figures embodying collective anxieties or vulnerable beings whose suffering heightens the audience’s emotional response [15,16]. Dogs occupy an especially ambivalent place here—culturally coded as loyal companions embedded in domestic intimacy, they become deeply unsettling when that trust is inverted, whether through contagion (*Cujo*—1983), metamorphosis (*The Thing*—1982), or uncanny behavioral disruption. Most scholarship on animals in horror has focused on these dynamics of inversion and monstrosity [17,18,19,20]. Far less attention has been given to films not staging the animal as a threat but trying to show the world from its perspective. This raises a distinct set of questions—not about how animals disrupt human order, but about whether cinema can adapt its language to non-human perception. And these questions carry weight when the animal involved is one with which audiences already share an intimate bond [21].

Among companion species, dogs occupy a uniquely significant position. Over the past several decades, their cultural status has undergone a profound transformation in Western societies: from a working animal or household to a family member, an emotional companion, and a sentient being whose capacity to feel makes it a subject of moral concern. This shift is reflected in the expansion of veterinary behavioral medicine [22], the growth of attachment research in human–dog dyads [23,24], the development of welfare-oriented legislation, and a public sensitivity to animal suffering that extends well beyond physical harm. The dog, more than any other domesticated species, is where evolving societal attitudes toward animal subjectivity are most visibly played out. Cinema both reflects and accelerates this process: the way dogs are filmed, respectively as props, as performers, as characters, or as subjects, mirrors how a given society conceives of them and, in turn, shapes public expectations about how they should be treated [21]. The relationship between society and its dogs and the relationship between cinema and its animal subjects are not parallel but entangled: each continuously reshapes the other.

This article examines how this entanglement works in practice. Does the contemporary human–dog relationship—in which dogs are increasingly recognized as sentient family members—reshape cultural production, and can cultural production reshape that relationship in return? *Good Boy* [25] provides an unusually dense case study. *Good Boy* follows Indy, a dog who moves with his owner Todd into an isolated, inherited family house in rural New Jersey. Indy soon senses a malevolent supernatural presence that the increasingly frail Todd cannot perceive, and the dog grows uneasy as he watches the threat close in on his owner. Told entirely from the dog’s point of view, the film gradually reveals that the haunting figures Todd’s terminal illness—a danger Indy can sense but is powerless to stop. Unusually, the protagonist is not a trained animal actor but the director’s own untrained family dog, filmed in the director’s own home. In *Good Boy*, the dog occupies three roles simultaneously: a real animal whose welfare is at stake during production, the sole protagonist through whose eyes the story is told, and the figure onto whom audiences project their emotions. The analysis is organized around three empirical axes. First, a qualitative analysis of filmmaker interviews, documents the production conditions and asks whether the filmmaker’s relationship with his dog tangibly shaped practice. The second, a behavioral coding of the dog’s on-screen signals, asks whether signs of stress are visible despite the filmmaker’s welfare intentions. The third, a thematic analysis of audience reviews, asks whether viewers leave the film with a changed understanding of dogs or simply with entertainment. Together, these three axes trace the circuit through which the human–dog relationship enters a cultural object and, potentially, returns to society transformed.

## 2. Materials and Methods

### 2.1. Qualitative Analysis of Filmmaker Interviews

In order to investigate the production conditions, directorial intentions, and cinematographic strategies underlying *Good Boy* [25], a corpus of five publicly available interviews with the filmmaker was assembled [26,27,28,29,30], all consulted on the 21st of May 2026, drawn from film industry and cultural publications.

A reflexive thematic analysis following Braun and Clarke’s (2006) [31] six-phase framework was conducted with AI assistance, adapting the procedure described in Hoummady et al. (2025) [32]. Claude Sonnet 4.6, accessed via the Abacus.AI platform with the “Disable Memories” setting enabled, was used to generate an initial codebook from the interview transcripts, which were provided as PDF files alongside the foundational methodological article [31]. Two sequential prompts were used. The first instructed the model to conduct phases 1 through 5 inductively—without a pre-existing framework—and to output a structured codebook table (theme label, definition, sub-themes, initial codes, and verbatim supporting quotes with line references). The second instructed the model to produce the phase-6 analytical narrative from the codebook.

Prompt 1:

You are a qualitative research assistant. You have no prior knowledge of the researcher, their hypotheses, or their theoretical orientation. Below are transcripts of five interviews with filmmaker Ben Leonberg about his 2025 film Good Boy.

Conduct phases 1 through 5 of Braun & Clarke’s (2006) [31] reflexive thematic analysis. Let all themes emerge inductively from the data. Do not impose any pre-existing framework.

Output a structured codebook as a table with the following columns: Theme label | Theme definition | Sub-theme (if applicable) | Initial codes grouped under this theme | Verbatim supporting quote(s) with line reference respectively: Campione, 2026 [26]; Macaulay, 2025 [27]; Manery, 2025 [28]; McCormack, 2025 [29]; Strouse, 2026 [30].

Do not write a narrative report at this stage. Only produce the codebook table.

Prompt 2:

Based on the codebook you produced, now write phase 6: the analytical narrative report. For each theme, write 1–2 paragraphs that define the theme, explain its significance in relation to the data, illustrate it with verbatim quotes, and discuss connections between themes. Write in academic English suitable for a peer-reviewed journal in animal welfare and cultural studies. Do not use bullet points. Be concise.

The AI-generated codebook served as a structured starting point. All codes and supporting quotations were then reviewed against the original transcripts, verifying that each code was grounded in the source material and that no quotation had been misattributed or fabricated. Minor adjustments were made: code labels and theme boundaries were refined, and two codes were reassigned to different themes based on contextual reading. No themes were added or removed. The final thematic structure reflects an AI-assisted, human-validated analysis. A limitation of this approach is that it does not fully replicate the iterative, reflexive engagement with data that Braun and Clarke’s framework [31] treats as constitutive of thematic analysis; this is partially mitigated by the human audit but not eliminated and is discussed further in Section 4.4.

### 2.2. Behavioral Coding of Focal Sequences

#### 2.2.1. Material and Sequence Selection

The behavioral analysis is based on *Good Boy*, acquired legally by N.R. via Amazon Prime Video on 12 January 2026 (order no. D01-7182399-5253452). To enable frame-by-frame behavioral coding, sequences were extracted by screen capture of the streamed content, as direct file export was not technically available through the platform. Video brightness was adjusted uniformly across all clips using FFmpeg (version 8.0.1) to improve visibility of the dog’s body parts under the film’s low-light conditions, without altering playback speed or frame content.

Eleven sequences were selected through purposive sampling to represent contrasting narrative and affective contexts within the film. Each sequence was delimited based on scene boundaries identified through repeated viewing and labeled according to its dominant narrative content (Table 1). Because our material is the released film and not the raw rushes, every sequence reaches us already selected, edited and scored; diegetic affective category is therefore a property of the finished artefact, not a record of the dog’s on-set experience. This is a structural limitation of analyzing any distributed film, and it bounds every comparison that follows. Affective categories were assigned a priori based on the narrative function of each sequence within the film’s horror structure. Neutral sequences (*n* = 4) were defined as moments of domestic routine, outdoor exploration, or passive resting, devoid of supernatural or threat-related cues. Anxiety-inducing sequences (*n* = 7) were defined as scenes that embed canonical horror conventions—spatial disorientation, social isolation, confrontation with an unknown stimulus, or separation—as perceived within the diegetic context. The imbalance between categories (4 neutral, 7 anxiety-inducing) reflects the overall structure of the film, in which threat-laden sequences predominate; this asymmetry is acknowledged as a limitation of the comparative analysis and discussed in Section 4.4.

As the filmmaker interviews make clear (Section 3.1), many emotionally charged moments were constructed through post-production editing and sound design, using footage of the dog responding to mundane stimuli. Conversely, a narratively neutral sequence may have involved mildly aversive production conditions (unfamiliar equipment, repeated takes). A significant difference in stress indicators between categories would therefore indicate that the dog’s behavioral state covaries with the film’s horror register, but would not establish whether this reflects a genuine affective response to on-set conditions, a post-hoc editorial alignment between the dog’s spontaneous discomfort and scenes in which that footage was retained, or both. This interpretive constraint is inherent to any behavioral analysis of edited film rather than raw production footage.

#### 2.2.2. Behavioral Repertoire

Behavioral coding was performed using BORIS v9.4 (Behavioral Observation Research Interactive Software; [33]) with continuous focal sampling. A behavioral repertoire of 21 behaviors developed based on preliminary observations of the focal sequences and adapted from published canine behavioral repertoires and relevant papers [34,35,36,37,38,39]. Facial expressions (e.g., brow tension, pupillary dilation) were not included in the behavioral repertoire. This reflects a deliberate focus on whole-body behaviors robust to the film’s variable, frequently oblique and low-angle framing, and is not a consequence of the low-light conditions or the brightness adjustment.

Behaviors were organized into two mutually exclusive state-event blocs and a set of point events (Table 2). Within each bloc, exactly one state was always active; both blocs were initialized from the first coded frame of each sequence. The structure of the blocs is summarized in Table 3.

It is worth noting that lip licking, avoidance, and low tail posture are established behavioral indicators of negative affect in dogs. These behaviors increase significantly under both acute and chronic stress conditions and correlate with elevated salivary cortisol levels [39,40]. Lip licking has been further characterized as a stress sign produced during conflict or discomfort in interspecific interactions [41], while avoidance and tail tucking are recognized markers of fear in validated behavioral repertoires [42]. The co-occurrence of these signals strengthens the inference of a negative emotional state, though interpretation should account for situational context [39]. Also, wagging was coded solely as the presence of repetitive lateral tail movement, without inference of emotional valence; wagging direction (left- vs. right-biased [43]) was not coded, as horizontal shot flipping during editing makes on-screen lateralization an unreliable guide to the dog’s actual lateralization.

#### 2.2.3. Coding Procedure

All eleven sequences were coded from video under a continuous recording protocol: state events (locomotion, tail position) scored for their full duration, point events at their exact time of occurrence. Lip licking was scored only as a stress signal; tongue movements during food anticipation or consumption were not coded [41], which in practice concerned the meal-in-front-of-TV sequence. Note that low tail was initially coded as a state event; inspection revealed exclusively brief, transient occurrences inconsistent with a sustained postural state, and it was therefore recoded as a point event (onset only) prior to reliability assessment. Because our material is the released film rather than raw footage, we cannot rule out that this brevity partly reflects editing rather than the dog’s spontaneous behavior; onset was therefore retained as the only aspect of this behavior that can be scored independently of a duration editing may have altered.

Reliability was established in three steps reflecting the study’s progression. First, intra-observer reliability was assessed by having the single observer (N.R.) re-code all sequences in randomized order, blind to first-pass results. Second, inter-observer reliability was added by having a second observer (S.H.) independently code the full behavioral repertoire across all eleven sequences. For state events, agreement was evaluated using the intraclass correlation coefficient (ICC, two-way mixed, absolute agreement) on the proportion of visible time per state; for point events, using Cohen’s kappa on event occurrence within 3-s bins. A threshold of 0.80 was adopted throughout, following McHugh (2012) [44]. The inter-observer comparison for state events used the mean of N.R.’s two passes; for point events, an occurrence was counted for N.R. only when coded in both passes.

Agreement outcomes followed an a priori decision rule applied uniformly: behaviors reaching threshold were retained as coded; sub-threshold behaviors that were not a priori stress indicators were excluded; and sub-threshold a priori stress indicators (lip licking, avoidance, low tail; [39,40,41,42]), which could not be excluded given their centrality to the research question, were treated as evidence of an under-specified operational definition rather than as grounds for exclusion. All three fell below threshold at the bin level between observers (Section 3.2.1).

Third, following this rule, the definitions of the three indicators were refined through a consensus procedure: two authors (N.R. and A.B.) identified the sources of ambiguity. The refined definitions were documented and fixed before any re-coding and before any agreement or test statistic was computed on the new codings. The three behaviors were then independently re-coded across all eleven sequences by N.R. and A.B., both observers blind to each other’s codings. N.R. re-coded in full rather than reusing the first-pass codings. Because the refined definitions differ from those of the initial passes, the re-coded data constitute an independent measurement rather than a continuation; values are reported separately and are not directly comparable (Section 3.2.1).

#### 2.2.4. Handling of Visibility Constraints

The film’s cinematographic style (low-angle shots, moving camera, frequent reframing) meant that the dog was partially or fully out of frame for a substantial and variable proportion of each sequence. Two categories of non-observable time were distinguished: locomotion not visible (body out of frame or occluded) and tail not visible (tail occluded by framing, furniture, or body orientation). To ensure comparability across sequences, all indicators were computed relative to observable time: locomotion state proportions as percentages of locomotion-visible time, tail position proportions as percentages of tail-visible time, and point event rates as events per minute of locomotion-visible time or tail visible time.

#### 2.2.5. Statistical Analysis

Because this is a single-subject design (one dog, 11 sequences), the behavioral analysis addresses two questions.

First, are signs of stress visible at all, despite the filmmaker’s welfare intentions? With a single dog, a fixed stress threshold would be arbitrary so we describe how often the signals appear rather than testing them against a criterion. For each a priori indicator we report its rate (events per minute of visible locomotion time) and the number of sequences in which it occurs. We then asked whether the three indicators tend to peak together. Within each sequence, an indicator was flagged when its rate was higher than its median across the eleven sequences, and a sequence was called convergent when at least two of the three indicators were flagged at once, meaning several stress signals coinciding strengthens the inference of a negative emotional state [39].

Second, do stress signals appear more often in anxiety-inducing sequences than in neutral ones? Because sequences were labeled by their role in the story, not by what the dog experienced on set (the structural constraint established in Section 2.2.1), a difference could show that the retained footage aligns with the film’s horror scenes—not that filming caused the stress. We compared categories with exact permutation tests, testing the observed group difference against all C(11, 4) = 330 possible splits. Tests were one-sided for the three a priori indicators (lip licking, avoidance, low tail; [39,40,41,42]), low tail recoded as a point event beforehand (Section 2.2.3). Each indicator value is the consensus rate of the two re-coders (N.R. and A.B.; Section 3.2.1). Analyses used R v. 4.4.3 [45] with tidyverse [46]; reliability statistics (ICC and Cohen’s kappa) were computed with the irr package [47]; figures, ggplot2 [48].

### 2.3. Qualitative Analysis of Audience Reception

A similar AI-assisted thematic analysis was applied to audience reception data. User reviews of *Good Boy* were collected from Letterboxd.com using the browser extension Instant Data Scraper for automated data extraction. The adapted prompts are given below:

*Prompt 1*

*You are a qualitative research assistant. You have no prior knowledge of the researcher, their hypotheses, or their theoretical orientation. Below is a structured corpus of 242 audience reviews of the 2025 horror film Good Boy (dir. Ben Leonberg). Each review is identified by a unique ID, source platform, reviewer pseudonym, date, rating (where available), full text, number of likes, and language. Reviews are predominantly in English, with a minority in Portuguese, Spanish, German, and Russian; analyse all reviews in their original language.*

*Conduct phases 1 through 5 of Braun & Clarke’s (2006) [31] reflexive thematic analysis. Let all themes emerge inductively from the data. Do not impose any pre-existing framework.*

*Output a structured codebook as a table with the following columns: Theme label | Theme definition | Sub-theme (if applicable) | Initial codes grouped under this theme | Verbatim supporting quote(s) with review ID reference (*e.g.*, LB-014).*

*Do not write a narrative report at this stage. Only produce the codebook table.*

*Prompt 2*

*Based on the codebook you produced, now write phase 6: the analytical narrative report. For each theme, write 1–2 paragraphs that define the theme, explain its significance in relation to the data, illustrate it with verbatim quotes, and discuss connections between themes. Write in academic English suitable for a peer-reviewed journal in animal welfare and cultural studies. Do not use bullet points. Be concise.*

## 3. Results

### 3.1. Qualitative Analysis

The thematic analysis of the five filmmaker interviews produced seven themes, regrouped below into five subsections according to their conceptual affinity. Each theme is reported in full italics. The first subsection presents the film’s foundational premise (Section 3.1.1); the next two cover how welfare shaped the production model (Section 3.1.2) and how the canine perspective was constructed cinematographically (Section 3.1.3); the fourth groups the craft strategies used to build atmosphere (Section 3.1.4); and the fifth isolates the dog’s proximity to human suffering (Section 3.1.5), whose significance emerges only against the behavioral data (Section 3.2) and is developed in the Discussion.

#### 3.1.1. Canine Authenticity as Foundational Premise

***Theme 1: Radical Canine Authenticity***

*A foundational and recurrent commitment across all five interview sources is Leonberg’s principled refusal to anthropomorphize his canine protagonist, Indy. Positioning the film explicitly against the conventions of the Hollywood animal genre —“*This isn’t Air Bud or Lassie*”* [27]*—Leonberg constructs a cinematic dog defined not by trained performance or human-legible emotion, but by instinct, sensation, and the bounded reasoning of a domestic animal. The intellectual lineage he invokes is significant: the comparison to Jack London’s canine protagonists in White Fang and The Call of the Wild situates Good Boy within a longer literary tradition of naturalist animal representation, in which the animal’s interiority is rendered on its own terms rather than as a mirror for human sentiment.*

*This authenticating impulse is not merely aesthetic; it carries structural consequences for how the film generates meaning and affect. The very behaviors that define Indy as a recognizable household pet—staring at corners, barking at nothing—are the same behaviors Leonberg recruits as the film’s primary horror mechanisms. As he observes, “every dog owner has […] at some point been worried why their dog was barking at nothing or staring at an empty corner” [30]. The ordinary is thereby rendered uncanny without distortion. This theme establishes the epistemological ground from which all other thematic concerns radiate: because Indy is emphatically just a dog, the questions of what he perceives, what he cannot communicate, and what he cannot prevent acquire their full weight.*

#### 3.1.2. Production Conditions: Constraint and Family

***Theme 2: Constraint as Creative Method***

*If Theme 1 articulates the philosophical premise of the film, Theme 2 documents the material and logistical conditions under which that premise was realized. The production of Good Boy extended across approximately 400 shooting days over three years, structured almost entirely around Indy’s attention span—“*at tops like three hours*” per day [26] and his irreducible behavioral unpredictability. Rather than treating these conditions as deficits to be managed, Leonberg consistently reframes them as generative constraints that shaped the film’s form from the inside out. The analogy he draws is to documentary practice: “*We made the movie akin to how you might make a documentary. You’re following a subject over the course of years while they’re going about their life*” [28].*

*The consequences of this methodology are visible at every level of production. Storyboards functioned as flexible guides rather than fixed blueprints; co-writer Alex received frequent calls informing him that planned sequences would “*be a little bit different*” [30]. The crew was deliberately reduced to two people—Leonberg and his wife, Kari Fischer—because “*every extra person is another distraction*” [28]. What emerges from this theme is a model of filmmaking in which the collaborating non-human subject exercises genuine, if non-intentional, authorial pressure on the work. This connects directly to Theme 6, where the dog’s perspective functions not merely as a narrative device but as an active structural force: Indy does not merely appear on screen as a character; his embodied presence and behavioral limits determine what story can be told and how.*

***Theme 4: The Film as Intimate Family Archive***

*Good Boy is inseparable from the domestic biography of its makers. Leonberg and Fischer lived in the house where the film was shot; Indy is their own dog; the puppy footage that opens the film constitutes genuine home movies, captured before any cinematic intention existed—“*not thinking he would be in a movie*” [26]. This biographical saturation of the production is not incidental but constitutive: Indy behaves with naturalistic ease on set precisely because, as Leonberg notes, “*it’s Indy’s real house, which is why he acts like he lives there because he does” *[27]. Fischer’s contribution as producer and creative partner—she is a scientist by training, not a filmmaker—further underscores the familial rather than purely professional character of the collaboration: “*It was a really fun… it was a family project. You know, we’re married, he’s our dog*” ([30], Kari Fischer).*

*The film’s archival dimension extends beyond the production context into an explicit meditation on memory and loss. Leonberg frames the finished work as a form of permanent testimony to a beloved companion: “*if anyone’s deserving of it, it’s a family dog*” [30]. This impulse to immortalize Indy connects the personal and the universal: the film becomes both a private family document and a public cultural object through which audiences may recognize and mourn their own animal companions. This theme bridges naturally into Theme 5—the meditation on mortality—insofar as the film’s archival function presupposes the dog’s finitude. To make a permanent record of a living animal is already to acknowledge, however obliquely, that the animal will die.*

#### 3.1.3. Constructing the Canine Perspective

***Theme 6: Point of View as Formal and Narrative Engine***

*Point of view in Good Boy is not a stylistic choice applied to a pre-existing story but the constitutive condition from which both story and style are derived. Leonberg and his co-writer structured the screenplay around what Indy’s perspective could plausibly contain: “*We were frequently writing, ‘Indy’s point of view sees this.’ […] Noting the point of view and the perspective was a constant, important thing to have in every scene in the script*” [29]. This narratological commitment produces a distinctive information hierarchy in which the audience’s access to events is constrained by the dog’s sensory and cognitive limits—a form of restricted narration that amplifies tension precisely because the interpreter of events is least equipped to act on what he perceives. The formal result is a horror film whose dread is generated not by revelatory spectacle but by withheld understanding.*

*Leonberg is also conscious of the genre-subversive dimension of this structural choice. Familiar haunted-house conventions—the ominous corner, the creaking door, the unseen presence—are displaced from their conventional human anchor into the canine perspective, making them simultaneously recognizable and newly strange: “*I hope *Good Boy* from its outset feels like a classic haunted house movie, but totally new because we’re experiencing it through a dog’s eyes*” [30]. Significantly, Leonberg indicates that the exploration of POV will continue to define his practice beyond this film—“*I’m certainly going to continue to explore perspective and point of view as something that really powers a film in a very literal sense*” [26]—suggesting that Good Boy represents not an isolated experiment but the articulation of an evolving directorial identity organized around the generative possibilities of non-human focalization.*

***Theme 3: Cinematic Illusion and Audience Projection***

*Central to Leonberg’s account of how Good Boy produces its affective impact is a sophisticated deployment of the Kuleshov effect—the editing principle whereby a neutral expression acquires meaning from the shot that follows it. Leonberg is explicit and self-aware about this mechanism: “*The Kuleshov effect is where we see an expression of an actor with a blank face, and then, because of the point-of-view shot, we can feel what the actor is feeling*” [29]. Applied to a non-human animal, this principle takes on an additional epistemological dimension. Indy does not perform fear; he cannot. The emotion attributed to him by audiences is entirely a product of editorial and sonic construction. As Leonberg acknowledges with candour: “*In reality, he’s just looking at me standing behind the camera, like, What is Dad doing? With the filmmaking of the ominous corner and the music, the audience feels, Oh man, that dog is afraid. He’s not. The filmmaking is making you, the audience, afraid. You’re putting that onto him*” [29].*

*This theme carries significant implications for how the film theorizes the human–animal relationship at a representational level. The audience’s compulsion to project interiority onto the dog—to read concern, confusion, and fear into what Leonberg describes as a “*naturally occurring 1000-yard stare*” [27]—mirrors the broader cognitive and affective dynamic of companion animal ownership itself: the constant, structurally uncertain interpretation of non-verbal animal signals by humans who care deeply about their meaning. In this sense, the cinema screen becomes a controlled experimental space in which the audience’s relationship to animal subjectivity is made visible and, arguably, interrogated. This theme is in productive dialogue with Theme 1: it is precisely because Indy is presented as a real, unperforming dog that the audience’s projection onto him feels emotionally legitimate rather than manipulated.*

#### 3.1.4. Atmosphere and Craft

***Theme 7: Atmosphere Through Practical Craft***

*The final theme draws together the film’s technical and material strategies for constructing horror atmosphere without recourse to digital spectacle. Leonberg’s cinematographic approach is dictated in the first instance by the physical facts of his subject: wide-angle lenses deployed at floor level serve the canine POV while simultaneously producing the spatial distortion and vignetting appropriate to the genre. Keeping Indy’s eyes visible in frame is framed not as a technical preference but as an audience-engagement imperative—“*it gives the audience something to glom onto*” [28]—reinforcing the connection to Theme 3, in which the dog’s eyes function as the primary site of emotional projection.*

*Sound design occupies an equally central position in Leonberg’s account of atmosphere-building. Because all production audio was replaced in post-production, the film’s sonic world is entirely a crafted construction, assembled by sound designer Brian Goodheart from raw recordings of Indy’s vocalizations, which were then “bent” and repurposed to serve narrative and affective ends [26]. Weather and environment complete the atmospheric palette: Leonberg deliberately scheduled exterior shoots to coincide with fog, and deployed rain to produce what he describes as the emotionally legible image of a wet, suffering dog—“*wet dogs just look sad*” [27]. The practical, problem-solving character of all these solutions reflects the broader ethos identified in Theme 2, in which constraint and resourcefulness are not opposed but mutually constitutive. Taken together, these craft decisions articulate a coherent aesthetic philosophy: that horror atmosphere is most persuasive when it appears to arise from material reality rather than technological artifice, a principle that is, ultimately, inseparable from the film’s foundational commitment to the authenticity of its canine protagonist.*

#### 3.1.5. Mortality and the Limits of Canine Agency

***Theme 5: Helplessness, Mortality, and the Limits of Love***

*The film’s deepest emotional register concerns what Leonberg calls “*the painful space between knowing and powerlessness*” [28]: Indy can sense the approaching catastrophe that threatens his owner Todd, but he possesses no capacity to prevent it. His attempts to intervene—barking, whining, physically interposing himself—are structurally futile. This asymmetry between perception and agency, between loving and protecting, constitutes the film’s central tragic proposition. Leonberg situates this proposition within a broader theory of the ghost story as mortality allegory: “*I think that fundamentally all ghost stories are about mortality—what happens to us after we die, the inability to let go, or anxiety about what’s ‘beyond’*” [27]. The haunting in Good Boy is, on this reading, less a supernatural event than an exteriorization of the anxiety that accompanies loving a mortal being—or being loved by one.*

*Leonberg’s account also introduces an ethological dimension that deepens the theme considerably. He recounts a piece of hunting lore concerning mortally wounded animals instinctively seeking cold, wet ground in which to die—“*the mud made dying hurt less*” [27]—and incorporates this image into the film’s supernatural motifs. The convergence of animal instinct, physical vulnerability, and the proximity to death articulated here recasts the dog not merely as a loving companion but as a creature whose entire sensory apparatus may orient it toward suffering and dying in ways unavailable to human perception. The question Leonberg poses—“*What would it be like for a dog who would die to protect the person he loves?” *[27]—is both the film’s narrative engine and its most ethically searching inquiry. This theme is closely intertwined with Theme 1: it is only because Indy is presented as a real animal, governed by instinct rather than heroic intention, that his powerlessness reads as genuinely tragic rather than dramatically convenient.*

Together, these seven themes describe a production in which the dog’s welfare and his role as narrative subject are tightly linked: the filmmaker’s relationship with his animal shaped both the material conditions of filming (Themes 2, 4, 7) and the formal commitment to a canine point of view (Themes 1, 3, 6). This account, however, remains the filmmaker’s self-report; stated welfare intentions are not in themselves evidence of the dog’s affective state. Their confrontation with the behavioral coding (Section 3.2) is therefore required before interpretation and is taken up in the Discussion (Section 4).

### 3.2. Behavioral Indicators of Stress in Focal Sequences

#### 3.2.1. Intra- and Inter Observer Reliability

Table 4 reports intra- and inter-observer agreement side by side for each behavior. State events were evaluated by ICC, point events by Cohen’s kappa, against a common 0.80 threshold [44].

Two patterns emerge (Table 4). State events were generally robust across both schemes, with one telling asymmetry: between observers, agreement on locomotion occlusion degraded while agreement on tail occlusion improved—observers concurred on when the tail was unscorable but less on when the dog was out of frame. Point events show the central result: agreement was systematically lower between observers than within, and the drop was steepest precisely for the a priori stress indicators—most starkly for avoidance, which fell from excellent within-coder agreement (κ = 0.831) to below criterion between coders (κ = 0.392). Behaviors resting on near-zero or single-event variance (startle, body shake, human contact, yawning) reflect prevalence matching rather than discriminative reliability and are not interpreted.

The divergence concentrated on the indicators most central to the research question. This located the difficulty in cross-observer reproducibility rather than in single-coder stability, and motivated the definitional refinement and independent re-coding of the three indicators (Section 2.2.3).

Re-coding under refined definitions. The three indicators were re-coded under operational definitions clarified through consensus (Section 2.2.3). Lip licking was afterwards defined as a brief tongue movement across the lips or nose, excluded whenever food was visible on screen; crucially, each individual tongue movement was scored as one event, so that successive licks counted separately rather than as a single bout. Avoidance was defined as a rapid directional movement away from an identified stimulus (a human movement, a sound, or an object). Low tail was defined as the tail held below the horizontal line of the back, pressed against or tucked between the hind legs.

Under these definitions, inter-observer agreement between N.R. and A.B. reached or exceeded the 0.80 threshold for all three indicators: κ = 0.915 [0.886, 0.943] (lip licking), κ = 1.000 [1.000, 1.000] (avoidance), and κ = 1.000 [1.000, 1.000] (low tail). The disagreement seen between N.R. and S.H. therefore stemmed from under-specified definitions rather than from an intrinsic limit on coding these behaviors reliably. These values derive from definitions differing from those of the initial passes and are an independent measurement, not directly comparable to the figures above.

#### 3.2.2. Behavioral Profile Across Sequences

Throughout this section, “neutral” and “anxiety-inducing” denote positions within the film’s narrative structure rather than states of the dog: a neutral sequence is one without horror cues, defined only by contrast with the anxiety-inducing ones (Section 2.2.1). The film’s horror register therefore frames the whole analysis, which moves from a general behavioral profile (this section) to the three a priori stress indicators (Section 3.2.3) and finally to a formal comparison between categories (Section 3.2.4).

Locomotion was dominated by stationary postures across all sequences (Table 5). Walking and trotting/galloping appeared mainly in the three sequences involving outdoor or open-space movement (walk in the forest, house exploration, garage exploration), and trotting/galloping peaked in the single outdoor neutral sequence (walk in the forest, 38.2%). In several sequences, the dog was stationary for the entire visible locomotion time, so walking and trotting/galloping are reported as dashes (no visible occurrence). Tail wagging, when the tail was visible, was recorded in seven sequences and spanned both categories, with no systematic association with affective context.

#### 3.2.3. Convergence of a Priori Stress Indicators

Narrowing to the three pre-specified stress indicators (lip licking, avoidance, low tail), at least one was observed in nine of the eleven sequences (Table 6). Lip licking was the most widespread, recorded in eight sequences; avoidance occurred in five sequences (arrival at new house, car neutral, generator setup, sleepwalking, shower scene); low tail in a single anxiety-inducing sequence (arrival at new house). This single occurrence directly coincided with an acute acoustic startle—the owner dropping a metal object (a padlock and chain)—consistent with a punctual reaction to an identifiable stimulus rather than a sustained anxious state, and in line with its brief, transient character (Section 2.2.3).

Convergence of two or more indicators above the corpus median occurred in three anxiety-inducing sequences (arrival at new house, generator setup, shower scene) and in one neutral sequence (car neutral). No sequence showed all three simultaneously. The most convergent anxiety-inducing profiles, the shower scene and generator setup, correspond to the sequences with the strongest horror conventions, strengthening the inference of a negative emotional state beyond what any single indicator would support [39]. Notably, the tail was not visible during the shower scene, so low tail could not be scored there; its zero value reflects non-observability rather than true absence. The car neutral sequence, however, also reached convergence: it combined an elevated lip-licking rate (6.67 events/min) with avoidance (2.22 events/min), consistent with the documented aversive potential of vehicle confinement [49]. Because the corpus median is zero for avoidance and low tail, convergence for these indicators reflects mere presence rather than relative intensity, and the appearance of a convergent neutral sequence tempers any clean separation between categories.

This concentration of convergent signals, present mainly but not exclusively in anxiety-inducing sequences, is so far only a description; whether the difference between categories exceeds what random assignment of sequences would produce is tested next.

#### 3.2.4. Comparison Between Affective Categories

**Figure 1:**
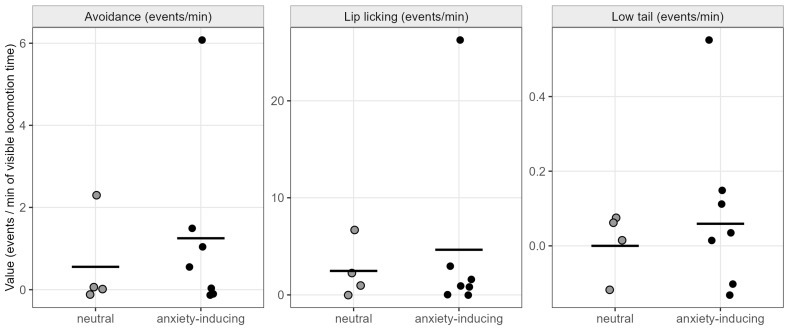
Distribution of a priori stress indicators by sequence category. Each point represents one sequence (grey = neutral, black = anxiety-inducing). Horizontal bars indicate group means.

The descriptive concentration just reported was then tested formally: does the difference between neutral and anxiety-inducing sequences exceed chance? Exact permutation test results for the three a priori stress indicators are summarized in Table 7 and visualized in Figure 1. Under the Stage 2 re-coding, none of the three indicators differed significantly between categories. Lip licking was numerically higher in anxiety-inducing sequences (4.65 vs. 2.47 events/min) but did not reach significance (*p* = 0.524), largely because the elevated rate in car neutral (6.67 events/min) inflated the neutral mean while the shower scene (26.3 events/min) acted as an extreme value within the anxiety-inducing group. Avoidance was also numerically higher in anxiety-inducing sequences but did not lead to a significant result (1.25 vs. 0.556 events/min, *p* = 0.379). Low tail occurred in a single sequence and was too rare to support any inferential comparison (*p* = 0.636).

These results led us to reframe the permutation test as exploratory and descriptive rather than confirmatory (Section 2.2.5). The inferential weight of the behavioral axis therefore rests not on any single category comparison but on the convergence of indicators within the most horror-laden sequences (Section 3.2.3), a pattern that both observers detected independently and that does not depend on the marginal between-category differences tested here.

### 3.3. Audience Reception

The thematic analysis of 242 Letterboxd reviews produced seven themes. These are reported below, grouped into three thematic clusters according to their relevance to the study’s central question—whether the film transforms the way audiences relate to dogs. Two additional themes—a recurrent tension between admiration for the film’s premise and dissatisfaction with its execution at feature length (Theme 2), and a register of self-aware humor, and reviews written in performative dog voice (Theme 6)—are not developed here but are noted as evidence of the evaluative diversity of the corpus.

#### 3.3.1. The Real Dog Eclipses the Fictional Threat

*Theme 5: Protective Anxiety over Dog Welfare*

*A distinctive finding is the extent to which concern for Indy-as-real-animal—rather than Indy-as-character—restructured the horror experience. The film’s most affectively charged scene was Indy being left outside in the rain (LB-021; LB-049; LB-162), generating more emotional intensity—expressed in capitalised, punctuation-heavy prose—than any horror set piece. Anger at the fictional owner’s neglect was itself experienced as dread (LB-001; LB-074), inverting the intended affective economy in ways that carry implications for both horror filmmakers and animal welfare scholars.*

*Theme 1: Indy as Exceptional Performer—The Canine Star*

*The corpus’s dominant theme is the construction of Indy as a performer of exceptional, institutionally under-recognised merit. Reviewers mounted sustained advocacy for Academy recognition of animal performance (LB-043; LB-079), invoking comparisons to Messi in Anatomy of a Fall (LB-047) and Daniel Day-Lewis (LB-005). Irony here functions as a culturally legible mode of sincerity within Letterboxd’s vernacular rather than a deflection of evaluative seriousness. Several reviewers resisted the claim that Indy ‘*didn’t know he was acting*’ framing such dismissiveness as an affront to the dog’s talent (LB-054). Rating inflation attributable solely to Indy’s presence (LB-084; LB-157) suggests his charisma operates as an independent evaluative variable, decoupled from assessments of the film as a whole.*

Across these two themes, reviewers engaged with Indy primarily as a real animal rather than as a fictional character. Concern for his welfare and recognition of his labor were recurring topics; no review in the corpus referenced any specific behavioral indicator of stress.

#### 3.3.2. Emotional Projection and Anthropomorphic Reading

*Theme 4: Emotional Resonance and the Human–Animal Bond*

*A substantial portion of the corpus records unexpected affective intensity—crying, prolonged emotional carry-over, the film* weigh on the soul*’ (LB-240)—rooted in identification with the dog-owner relationship and projection of personal pet histories (LB-019; LB-118). The film’s horror marketing was partially misleading for these viewers: one Portuguese-language reviewer described it as ‘a heavy drama using the aesthetics of horror as a scaffold’ (LB-186). The most-cited line—‘*You’re a good dog. But you can’t save me*.’ (LB-077; LB-228)—anchors a widely shared allegorical reading in which the supernatural entity figures Todd’s illness (LB-178), repositioning Indy’s protectiveness as a meditation on the limits of interspecies care. This connects recursively to Theme 5: audience anxiety for Indy mirrors Indy’s anxiety for Todd, producing a shared posture of helpless love.*

*Theme 3: Formal Innovation and the Dog’s Perceptual World*

*The film’s sustained adoption of a dog’s-eye perspective was its most consistently praised formal achievement. Reviewers articulated its horror logic through the folk belief that dogs perceive presences invisible to humans (LB-020; LB-207), identifying this as the film’s most substantive contribution to the haunted house subgenre (LB-115). Sound design and cinematography were cited as mutually reinforcing elements (LB-156; LB-165), though the slow-burn pace divided opinion: genre-literate viewers schooled in experimental horror—referencing Skinamarink and Mike Flanagan (LB-114; LB-142)—praised its restraint, while others experienced it as pacing failure, directly connecting this theme to the gimmick critique of Theme 2.*

The dominant affective register across both themes was identification with the dog–owner relationship. Reviewers interpreted Indy’s on-screen proximity to Todd as devotion, and praised the canine perspective as a formal achievement, without distinguishing between the dog’s spontaneous behavior and its editorial construction.

#### 3.3.3. Welfare as an Emerging Aesthetic Criterion

*Theme 7: Appreciation of Independent Production Context*

*Knowledge of the film’s production circumstances—four years, approximately 400 shooting days, a $70,000 budget, and a director working with his own untrained family dog—systematically inflected quality judgements upward. Reviewers invoked this context as a mitigating lens on formal shortcomings, treating logistical patience as an aesthetic virtue (LB-033; LB-179). The post-credits making-of sequence was cited as transformative, retrospectively elevating the viewing experience (LB-099; LB-212). The director-as-dog-owner framing positioned the film as a ‘love letter’ to Indy (LB-008), lending it emotional authenticity grounded in a real interspecies bond. The accompanying ‘nepobaby’ discourse (LB-051; LB-148) operates in productive tension with Theme 1’s meritocratic framing, revealing an audience negotiating competing models of animal labor—natural talent, family member, and ethical worker—whose coexistence within single reviews attests to the unresolved cultural negotiations surrounding animal-centred filmmaking.*

A subset of reviewers explicitly integrated production conditions—budget, timeline, the filmmaker’s relationship with his dog—into their aesthetic evaluation of the film.

## 4. Discussion

This article asked whether the growing recognition of dogs as sentient family members reshapes cultural production, and whether cultural production can, in turn, reshape the human–dog relationship. *Good Boy* provided a uniquely dense case because the dog simultaneously occupies three roles—a real animal whose welfare is at stake, the sole narrative subject, and the figure onto whom audiences project their emotions. Each empirical axis addressed one of these roles. Together, they reveal a consistent pattern: the contemporary human–dog relationship does enter cultural production and does reshape it—but incompletely, and with consequences that none of the actors involved fully control.

### 4.1. From Relationship to Production: Welfare as a Creative Force and Its Limits

The first axis asked whether the filmmakers’ relationship with their dog tangibly shaped production practice. The entire production model—400-day timeline, the two-person crew, the use of the dog’s own home, the absence of coercive training—suggests an organization structured around Indy’s welfare needs. Unlike most productions, the filmmaker was working with his own dog, a familiarity that enables an individualized understanding of the animal’s needs. These choices align with contemporary frameworks that define animal welfare not merely as the absence of physical harm but as encompassing affective experience, behavioral expression, and agency [2,8], and represent a concrete translation of the bond between a dog and his owners into professional practice. *Good Boy* demonstrates that the cultural status of dogs as family members can restructure the conditions under which they participate in media production.

However, the behavioral data complicates this picture. Good intentions are not welfare evidence [50], and Leonberg’s account remains an unverified self-report. The behavioral coding shows that Indy was not uniformly at ease. The sequences with the most convergent stress profiles—shower scene, generator setup, arrival at new house— are also those with the strongest horror conventions. In each, multiple indicators co-occur (lip licking, avoidance, or low tail depending on the sequence)—a pattern difficult to attribute to incidental conditions [39,40,41,42]. None of the indicators reached statistical significance once the data were independently re-coded by both observers (Section 3.2.4), and the inference therefore rests not on any single test but on this convergence: two behaviorally independent stress channels concentrating in the most horror-laden sequences, detected independently by both coders. The dog’s stress and the film’s horror scenes are more closely linked than the filmmakers’ reassurances about welfare would suggest.

The car neutral sequence reveals a different facet of the same problem, and a more pointed one. Coded as neutral on narrative grounds, it was nonetheless one of the convergent sequences, combining a lip-licking rate exceeding every anxiety-inducing sequence except the shower scene with the corpus’s second-highest avoidance rate, likely reflecting the well-documented aversive potential of vehicle confinement [49]. A scene the audience reads as uneventful is not necessarily one the dog experienced as comfortable. This raises a deeper paradox for any realist approach to animal filmmaking: if authenticity requires the dog to display genuine behavioral indicators of distress, then the pursuit of realism creates an inherent tension with welfare. The alternative, meaning constructing the appearance of distress entirely through post-production tools such as sound design, editing, and visual effects would protect the animal but undermine the very authenticity the filmmaker seeks. *Good Boy* relies on both strategies without fully resolving the tension between them, and the question of where the ethical boundary lies between capturing real behavior and manufacturing it in post-production remains open.

These results produce a paradox at the heart of the relationship-to-production circuit. The sequences where Indy shows genuine stress are also those where his behavior is most ethologically legible—a real dog communicating real discomfort through scientifically documented signals. The film’s welfare deficit and its representational authenticity share the same source. Documentary cinema has long faced an analogous tension: it presents itself as a faithful record of the natural world, yet the authentic emotion it puts on screen is frequently manufactured. Scenes staged through baiting or chumming, and narration that overlays anthropomorphic affect in place of accurate ethological description [14]. What audiences receive as observation is often construction, blurring the documentary’s implicit claim to show things as they are [51]. What distinguishes the animal case is the absence of consent, although recent work in animal welfare has begun to explore forms of animal assent, such as opt-in/opt-out protocols and consent training [22]. One actionable step would be to embed an independent welfare professional—a certified veterinary behaviorist or applied animal ethologist—within animal-centric productions, with the authority to pause or modify filming. Such oversight could be triggered by predefined behavioral thresholds rather than left to directorial judgement: the co-occurrence of stress indicators we observed in the shower scene (lip-licking peaking at high rate alongside avoidance) illustrates the kind of convergent micro-signal that could serve as a stop criterion, though an operational threshold would need to be established on multi-subject data rather than derived from a single case. This would shift welfare from a matter of filmmaker goodwill to a monitored, enforceable condition of production. A human subject can agree to the terms of observation; a dog cannot. This shifts the ethical burden entirely onto producers and regulators—and current frameworks remain oriented toward preventing overt physical harm rather than monitoring affective states [9,10]. But even if such frameworks were in place, *Good Boy’s* artisanal model—a two-person crew, a 400-day timeline, the dog’s own home—cannot scale to industrial production, and what welfare-responsible animal filmmaking looks like under commercial conditions remains an open question.

### 4.2. From Production to Representation: What the Canine Perspective Reveals and Where It Breaks Down

The second axis asks whether the human–dog relationship, once it enters production, can generate a representation of canine subjectivity that holds—or whether the attempt reveals where the cultural desire to understand dogs outpaces our capacity to show their experience.

That a filmmaker would spend three years trying to show the world through a dog’s eyes—and that audiences would care—is itself evidence of where the human–dog relationship now stands. The attempt matters, even where it falls short.

*Good Boy* retains several conventions of horror cinema: the haunted house, the isolated setting, the slow build toward confrontation. But it departs from the genre in ways that are directly tied to the dog’s presence. Indy occupies a hybrid narrative position—he perceives the threat before the human characters, yet he cannot act on what he knows. This makes him a witness, not a hero, and the distress this generates in the audience is different from conventional suspense: it comes from watching someone who cannot help.

Visually, the film pushes a familiar device—low-angle framing—further than the genre typically requires. Human faces are often excluded, reducing characters to legs, hands, and partial silhouettes. This removes the viewer’s main tool for reading emotion and forces a state of uncertainty that, while effective as horror, originates in the decision to film from the dog’s physical position. Similarly, the threat remains structurally opaque for most of the film, accessible only through Indy’s behavioral reactions—his gaze, his posture, his avoidance. This recalls a wider tendency in recent horror to displace knowledge from the visual to the sensory register, denying the audience the reassurance of seeing and so forcing a different relationship to what threatens [52]. When the threat is finally revealed, it takes the form not of a conventional antagonist but of a metaphor for terminal illness. Even in resolution, the source of danger is never something a dog could understand or prevent—only something he can sense. This sustains the canine perspective while departing significantly from genre norms, in which the threat is eventually made visible and confrontable.

What is striking is that some of these formal innovations were not planned. *Good Boy*’s slow rhythm, its extended gazes, its long takes—all emerged from the practical impossibility of scripting a real dog. Each shot required waiting for a usable behavioral moment, and this constraint produced a pace that is more observational and documentary-adjacent than most horror films. The formal novelty is real, but largely involuntary. This has a broader implication: when a filmmaker takes animal subjectivity seriously enough to let it constrain production, it disrupts established conventions and generates something genuinely new. The relationship between the filmmaker and his dog did not just shape logistics; it reshaped the film’s cinematic language.

Yet the attempt to represent canine subjectivity breaks down in several places. The concept of Umwelt—the species-specific perceptual world each organism inhabits [53]—offers a useful framework for identifying these limits, because it foregrounds the sensory channels through which a given species constructs its experience of the world. Dogs experience the world primarily through smell and hearing: roughly 250 million olfactory receptors against our five million [54,55], and hearing that extends to 47 kHz [56,57]. *Good Boy* constructs its canine perspective exclusively through vision—low framing, spatial restriction, exclusion of human faces. The sound design is built entirely for human ears: its dissonances, silences, and tonal shifts are calibrated to frighten a human audience, with no attempt to evoke the canine auditory world.

Some of these gaps are presented as technical constraints, yet the limitation is less technical than perceptual. Olfaction could be staged as some 4DX venues already diffuse scents synchronized to on-screen action. Sounds outside the range of human hearing could at least be signaled, if not reproduced. The real difficulty is not generating a stimulus but conveying the animal’s sensory world: It seems difficult to recreate what an odor is to a dog, whose olfactory perception is structured in ways largely inaccessible to us. Other gaps, however, are choices. Dichromatic color vision [58] could have been achieved through simple color grading. And the near-systematic exclusion of human faces, while highly effective as a horror device, is paradoxical from an ethological standpoint: dogs actively monitor human faces, show a left gaze bias toward them [59], and discriminate between facial expressions [60], and engage in mutual gaze that activates oxytocin pathways in both species [61]. A canine perspective that removes human faces is, for a dog, less canine than more.

A further tension concerns narrative focalization. The canine perspective holds when the horror register requires perceptual restriction—the viewer sees only what the dog sees. But whenever the plot needs verbal information—a medical consultation, a phone call—the spectator gains access to language that a dog could not process. The canine point of view works as a narrative device, activated for tension and suspended for exposition, rather than as a consistent formal commitment.

The result is a human perspective lowered to canine height—closer to a dog’s world than most films attempt, but still fundamentally shaped by human perception. Subjective experience is tied to the organism’s own perceptual apparatus: cinema can show where a dog looks, but not what looking is like for a dog. Weber and Hawranke’s (2024) [62] analysis of the video game *Stray* offers a useful contrast: *Stray* maintains a third-person perspective on its animal protagonist, preserving the opacity of non-human experience rather than claiming to inhabit it. *Good Boy*’s first-person approach sets a higher standard—and makes its own limits more visible.

These limits are not failures of one film. They mark the point where the cultural desire to understand dogs—a desire that is itself a product of the changing human–dog relationship—exceeds what current representational tools can deliver.

### 4.3. From Representation to Audience: Does the Film Reshape the Relationship?

The third axis asked whether the film transforms the way audiences relate to dogs, thereby completing the circuit from relationship to production to society. The audience data suggests a partial and ambivalent answer.

On one hand, the reviews reveal a shift in what audiences attend to. Concern for Indy as a real animal, not as a fictional character, restructured the horror experience for a significant portion of viewers. The film’s most affectively charged moment was not a horror set piece but Indy being left outside in the rain; anger at the fictional owner’s neglect was experienced as dread. For these viewers, the fear of a real dog being harmed overrode the fear of a fictional ghost. The human–dog bond proved more emotionally powerful than the horror narrative built around it. Similarly, knowledge of the production conditions—the three-year timeline, the minimal budget, the filmmaker working with his own dog—systematically inflected quality judgments upward. For a portion of the audience, how a film treats its dog became part of what makes it a good film. This points to an emerging aesthetic criterion rooted in welfare values.

On the other hand, the dominant mode of audience engagement remains anthropomorphic projection. Viewers read Indy’s proximity to Todd as loyalty and his behavior as devotion. The framing is emotionally powerful, but ethologically misleading. Dogs do form selective attachment bonds with their caregivers including proximity-seeking, separation distress, secure-base effects [23,24,63]. But attachment is not loyalty. Loyalty implies conscious commitment; the behaviors the film presents as devotion are better understood as products of a bonding system shaped by domestication. Framing them as heroic choice reinforces anthropomorphic readings that can distort public understanding of what dogs need [13]. Worse, this framing hides a welfare problem: a dog staying close to an unpredictable caregiver in a threatening environment is not demonstrating devotion—it is likely exhibiting coping strategies or submissive behaviors linked to significant stress [39,64]. The loyalty narrative makes this invisible.

Strikingly, not a single review in the corpus mentions nose licking, gaze aversion, or any other subtle indicator of stress. Yet these signals are visible in the film. Where the audience sees a devoted dog, behavioral analysis sees a dog in discomfort. Observers read most reliably the emotion a dog displays most overtly, and that emotion is happiness: it is correctly identified around 88% of the time [65]. Negative states fare far worse—even fear, an overt emotion, is recognized only about 45% of the time and is routinely confused with surprise [65]. Subtle, early signals of stress are missed almost entirely [66]. Nose licking, for instance, is identified as a stress indicator by fewer than 5% of owners [67]. Owners also tend to overestimate their ability to read canine behavior [65] and everyday familiarity with a pet does not, by itself, yield accurate detection of early stress signals without explicit education [67]. The film therefore does two things at once: its editing reinforces anthropomorphic projection, while its unscripted footage records authentic behavioral material that could undermine it. It does reach its audience and shift how they relate to dogs—but what they take away is filtered through anthropomorphic projection rather than ethological understanding, because most viewers cannot read what the dog is communicating.

### 4.4. Limitations

This is a single-case, mixed-methods study; its value lies not in generalizability but in the convergence of three independent data sources on the same animal in the same production. The eleven coded sequences are a purposive sample of the first half of the film, and the imbalance between neutral (*n* = 4) and anxiety-inducing (*n* = 7) categories reflects the film’s own structure. The car neutral sequence confirms that diegetic classification does not reliably map onto affective experience, and this is the primary interpretive boundary of the behavioral axis. Because the analysis is conducted on a finished, edited film, the relationship between what is shown and what the animal experienced cannot be recovered by analytic means; only access to the unedited rushes would resolve it. This is not a limitation peculiar to the present study but a constraint that any investigation of animal welfare based on finished cultural products must confront. Relatedly, wagging was scored only as present or absent: although its lateralization carries affective valence [43], it could not be assessed here, since horizontal shot flipping during editing renders on-screen orientation an unreliable record of the dog’s actual movement. This is a further consequence of working from a finished film rather than raw footage.

The filmmaker interviews are publicly available and self-selected; omissions are not detectable. The AI-assisted thematic analysis prioritizes transparency and reproducibility at the cost of the reflexive engagement Braun and Clarke’s (2006) [31] framework treats as constitutive—a trade-off we chose deliberately and partially compensated through a complete human audit of every code and supporting quotation against the source material. This audit identified a single contextual misreading—references to Does the Dog Die? initially coded by the AI as concern for the real animal, whereas they in fact reflected anxiety about the character’s fate within the narrative—which was corrected accordingly; no other misattribution was found. The audience corpus (242 Letterboxd reviews) is platform-specific; the observed patterns are internally consistent but would require experimental testing to establish broader prevalence.

Rather than weaknesses of the present work, these constraints mark the directions along which its triangulated, single-case approach can be scaled—to larger corpora, multiple coders, and other species—in future research.

## 5. Conclusions

This study is novel in situating itself at the intersection of ethology and cultural studies—bringing quantitative behavioral coding together with the qualitative analysis of filmmaker discourse and audience reception, applied to a single film animal read at once as a behaving subject and as a cultural figure. Through this approach, it traced the circuit by which the human–dog relationship enters cultural production and returns to society. At each stage, the translation proved real but incomplete: the filmmaker’s bond with his dog restructured production, yet stress signals persisted; the film tried to construct a canine perspective that generated formal innovation yet remained confined to human perception; audiences showed concern for the real animal yet read his discomfort as devotion. *Good Boy* reveals not the failure of one film but the current state of a cultural transition—a society that has moved beyond treating dogs as props but lacks the tools to act on its own values. Three needs emerge: independent welfare monitoring during production, representational strategies beyond visual, and public education in canine body language.

## Figures and Tables

**Table 1:** Extracted sequences, durations, and affective classification.

#	Sequence Label	Timecode	Duration	Category
1	Arrival at new house	00:07:45–00:10:34	2 min 49 s	Anxiety-inducing
2	House exploration	00:12:08–00:14:57	2 min 49 s	Anxiety-inducing
3	Walk in the forest	00:15:13–00:16:47	1 min 34 s	Neutral
4	Wall	00:18:43–00:19:12	29 s	Neutral
5	Car neutral	00:19:11–00:19:39	28 s	Neutral
6	Generator setup	00:20:38–00:21:44	1 min 06 s	Anxiety-inducing
7	Meal in front of TV	00:22:26–00:22:46	20 s	Neutral
8	Dog left alone	00:25:50–00:30:22	4 min 32 s	Anxiety-inducing
9	Sleepwalking	00:35:05–00:36:28	1 min 23 s	Anxiety-inducing
10	Garage exploration	00:38:22–00:40:17	1 min 55 s	Anxiety-inducing
11	Shower scene	00:42:02–00:42:44	42 s	Anxiety-inducing

**Table 2:** Behavioral repertoire of the 21 coded behaviors, organized into locomotion states, tail-position states, and point events, with operational definitions and sources.

Code	Behavior	Description	Source
Locomotion states (mutually exclusive, coded as continuous state events)			
k	Stationary	No visible body displacement—standing, sitting, or lying	—
m	Walking	Forward locomotion at moderate pace	—
t	Trotting/galloping	Fast-paced locomotion, including short leaps absorbed within the bout	—
b	Backing	Locomotion directed backward, away from a stimulus	—
c	Crawling	Forward locomotion in a lowered posture with the ventral surface close to the ground	—
z	Locomotion not visible	Dog occluded by a human passing in front of the camera during an ongoing locomotion bout. Default: stationary if the dog left the frame without identifiable cause	—
Tail position states (mutually exclusive, coded as continuous state events)			
q	Low	Tail held below the horizontal line of the back, pressed against or between the hind legs	[35]
h	High	Tail held above the horizontal line of the back	[35]
u	Neutral	Tail aligned with the hindquarter at or near the horizontal. Default when the dog is in a sitting posture; if lateral oscillatory movement clearly visible, code as wagging	[35]
w	Wagging	Repetitive lateral tail movements	[35]
x	Tail not visible	Tail occluded by framing, body position, or the dog being out of frame	—
Point events (instantaneous, scored at the moment of onset)			
l	Lip licking	Brief tongue movement across the lips or nose	[34]
e	Avoidance	Rapid directional movement away from a stimulus	[34]
j	Startle	Sudden whole-body flinch in response to an abrupt stimulus	[34]
p	Jump	The dog mounting onto or descending from an elevated surface, coded independently of the locomotion state in progress	—
v	Vocalization	Visible lip or jaw movement consistent with vocal production. Sound type not inferred (post-production audio track)	—
f	Object sniffing	Muzzle oriented toward and in proximity to a surface or object with visible nostril movement	[34]
r	Air sniffing	Muzzle raised with visible nostril movement, not directed at a specific object	[34]
s	Body shake	Rapid lateral oscillation of the whole body	[34]
y	Yawning	Wide opening of the mouth with visible inhalation	[34]
i	Human contact	Physical contact initiated by the dog toward a human	—

**Table 3:** Structure of the two state-event blocs (locomotion and tail position) and the point-event set, with their mutual-exclusivity constraints.

Bloc	Member States	Constraint
Bloc 1—Locomotion	stationary, walking, trotting/galloping, backing, crawling, locomotion not visible	Mutually exclusive; one active from frame 1
Bloc 2—Tail position	low, high, neutral, wagging, tail not visible	Mutually exclusive; one active from frame 1
Point events	lip licking, avoidance, startle, jump, vocalization, object sniffing, air sniffing, body shake, yawning, human contact	Instantaneous; scored at moment of onset

**Table 4:** Intra-observer (N.R., pass 1 vs. pass 2) and inter-observer (N.R. vs. S.H.) reliability per behavior. ICC for state events, Cohen’s κ for point events. Bold marks values meeting the 0.80 threshold. — = not assessable.

Behavior	Type	Intra-Observer	Inter-Observer
Stationary	State	**0.993 [0.976, 0.998]**	**0.989 [0.963, 0.997]**
Walking	State	**0.923 [0.572, 0.989]**	**0.865 [0.348, 0.980]**
Trotting/galloping	State	**0.989 [0.919, 0.999]**	**0.842 [0.107, 0.989]**
Backing	State	0.638 [−0.344, 0.942]	0.639 [−0.315, 0.942]
Crawling	State	— ^2^	— ^2^
Locomotion not visible	State	**0.931 [0.678, 0.984]**	0.537 [−0.090, 0.870]
Wagging tail	State	**0.974 [0.862, 0.995]**	**0.950 [0.691, 0.993]**
Neutral tail	State	**0.922 [0.644, 0.986]**	0.432 [−0.187, 0.859]
High tail	State	0.428 [−0.302, 0.886]	0.133 [−0.120, 0.727]
Tail not visible	State	0.553 [−0.009, 0.855]	**0.994 [0.978, 0.998]**
Lip licking	Point	**0.938 [0.913, 0.963]**	0.764 [0.591, 0.937]
Avoidance	Point	**0.831 [0.792, 0.869]**	0.392 [−0.091, 0.874]
Low tail	Point	**1.00 [1.00, 1.00]**	0.665 [0.011, 1.32] ^1^
Jump	Point	**0.932 [0.906, 0.958]**	0.393 [−0.089, 0.875]
Vocalization	Point	**0.856 [0.819, 0.892]**	0.135 [−0.207, 0.477]
Object sniffing	Point	0.772 [0.729, 0.816]	0.487 [0.173, 0.800]
Air sniffing	Point	0.664 [0.615, 0.713]	0.440 [−0.048, 0.927]
Startle	Point	— ^3^	— ^3^
Body shake	Point	— ^3^	— ^3^
Yawning	Point	— ^3^	— ^3^
Human contact	Point	— ^3^	— ^3^

^1^ Upper CI bound exceeds 1 owing to the normal approximation on very few events (two sequences for N.R., one for S.H.); interpreted with caution. ^2^ Crawling occurred in a single sequence, precluding ICC estimation. ^3^ Startle, body shake, yawning, and human contact occurred at near-zero or single-event prevalence: for these behaviors, at least one observer recorded zero occurrences in most sequences, making κ mathematically undefined.

**Table 5:** Locomotion-state and tail-position proportions per sequence, as percentages of visible time. Locomotion states are expressed relative to visible locomotion time (stationary + walking + trotting/galloping); wagging tail is expressed relative to tail-visible time (total time minus tail-not-visible). Cat. = affective category (N = neutral; A = anxiety-inducing). Dashes indicate states with no visible occurrence in the sequence.

Sequence	Cat.	Stationary	Walking	Trot/Gallop	Wagging Tail
Arrival at new house	A	81.9	16.6	1.6	32.3
House exploration	A	63.6	30.3	6.1	15.6
Walk in the forest	N	44.4	17.4	38.2	37.6
Wall	N	100	–	–	–
Car neutral	N	100	–	–	–
Generator setup	A	100	–	–	–
Meal in front of TV	N	100	–	–	100
Dog left alone	A	85.9	5.5	8.6	34.8
Sleepwalking	A	84.2	15.8	–	3.7
Garage exploration	A	65.8	25.6	8.5	12.1
Shower scene	A	100	–	–	–

Only states meeting the intra-observer reliability threshold are reported (Section 3.2.1).

**Table 6:** Rates of the three a priori stress indicators per sequence (Stage 2 re-coding, consensus of N.R. and A.B.), expressed as events per minute of visible locomotion time. Cat. = affective category (N = neutral; A = anxiety-inducing). Co-occ. = number of a priori indicators whose rate strictly exceeds its corpus-wide median (range 0–3); bold marks convergent sequences (≥2 indicators above median).

Sequence	Cat.	Lip Licking	Avoidance	Low Tail	Co-occ.
Arrival at new house	A	0.00	0.41	0.41	**2**
House exploration	A	0.00	0.00	0.00	0
Walk in the forest	N	0.96	0.00	0.00	0
Wall	N	2.24	0.00	0.00	1
Car neutral	N	6.67	2.22	0.00	**2**
Generator setup	A	2.95	1.48	0.00	**2**
Meal in front of TV	N	0.00	0.00	0.00	0
Dog left alone	A	1.61	0.00	0.00	1
Sleepwalking	A	0.89	0.89	0.00	1
Garage exploration	A	0.81	0.00	0.00	0
Shower scene	A	26.3	5.97	0.00	**2**
**Corpus median**		0.96	0.00 ^1^	0.00 ^1^	

^1^ For avoidance and low tail the corpus median is zero, so “above median” reduces to “present” (Section 2.2.5); convergence for these two indicators therefore reflects presence rather than relative intensity. Low tail is reported as an event rate, having been recoded as a point event (Section 2.2.3).

**Table 7:** Exact permutation test results for the three a priori stress indicators, comparing neutral (*n* = 4) and anxiety-inducing (*n* = 7) sequences (*C*(11, 4) = 330 permutations), based on the Stage 2 re-coding (consensus of N.R. and A.B.). Difference = mean anxiety-inducing minus mean neutral. All indicators tested one-sided (anxiety-inducing > neutral).

Indicator	Test	Mean Neutral	Mean Anxiety	Difference	*p*
A priori stress indicators					
Lip licking (events/min)	One-sided	2.47	4.65	+2.18	0.524
Avoidance (events/min)	One-sided	0.556	1.25	+0.694	0.379
Low tail (events/min)	One-sided	0.00	0.059	+0.059	0.636

A priori indicators tested one-sided (anxiety-inducing > neutral). No indicator reached the *p* < 0.05 threshold under the Stage 2 re-coding; the permutation test is therefore reported as exploratory (Section 2.2.5).

## Data Availability

The data supporting the findings of this study are available from the corresponding author upon reasonable request.
